# Aire Downregulation Is Associated with Changes in the Posttranscriptional Control of Peripheral Tissue Antigens in Medullary Thymic Epithelial Cells

**DOI:** 10.3389/fimmu.2016.00526

**Published:** 2016-11-23

**Authors:** Ernna H. Oliveira, Claudia Macedo, Cristhianna V. Collares, Ana Carolina Freitas, Paula Barbim Donate, Elza T. Sakamoto-Hojo, Eduardo A. Donadi, Geraldo A. Passos

**Affiliations:** ^1^Molecular Immunogenetics Group, Department of Genetics, Ribeirão Preto Medical School, University of São Paulo (USP), São Paulo, Brazil; ^2^Department of Pathology, Ribeirão Preto Medical School, University of São Paulo (USP), São Paulo, Brazil; ^3^Department of Biology, Faculty of Philosophy, Sciences and Letters of Ribeirão Preto, University of São Paulo (USP), São Paulo, Brazil; ^4^Department of Clinical Medicine, Division of Clinical Immunology, Ribeirão Preto Medical School, University of São Paulo (USP), São Paulo, Brazil; ^5^Discipline of Genetics and Molecular Biology, Department of Morphology, Physiology and Basic Pathology, School of Dentistry of Ribeirão Preto, University of São Paulo (USP), São Paulo, Brazil

**Keywords:** Aire, miRNAs, miRNA–mRNA interaction, promiscuous gene expression, medullary thymic epithelial cells, *in vivo* gene knockdown

## Abstract

Autoimmune regulator (Aire) is a transcriptional regulator of peripheral tissue antigens (PTAs) and microRNAs (miRNAs) in medullary thymic epithelial cells (mTECs). In this study, we tested the hypothesis that Aire also played a role as an upstream posttranscriptional controller in these cells and that variation in its expression might be associated with changes in the interactions between miRNAs and the mRNAs encoding PTAs. We demonstrated that downregulation of Aire *in vivo* in the thymuses of BALB/c mice imbalanced the large-scale expression of these two RNA species and consequently their interactions. The expression profiles of a large set of mTEC miRNAs and mRNAs isolated from the thymuses of mice subjected (or not) to small-interfering-induced Aire gene knockdown revealed that 87 miRNAs and 4,558 mRNAs were differentially expressed. The reconstruction of the miRNA–mRNA interaction networks demonstrated that interactions between these RNAs were under Aire influence and therefore changed when this gene was downregulated. Prior to Aire-knockdown, only members of the miR-let-7 family interacted with a set of PTA mRNAs. Under Aire-knockdown conditions, a larger set of miRNA families and their members established this type of interaction. Notably, no previously described Aire-dependent PTA interacted with the miRNAs, indicating that these PTAs were somehow refractory. The miRNA–mRNA interactions were validated by calculating the minimal free energy of the pairings between the miRNA seed regions and the mRNA 3′ UTRs and within the cellular milieu using the luciferase reporter gene assay. These results suggest the existence of a link between transcriptional and posttranscriptional control because Aire downregulation alters the miRNA–mRNA network controlling PTAs in mTEC cells.

## Introduction

Autoimmune regulator (AIRE) is a key protein that plays a role as a controller of the transcriptional expression of a large set of peripheral tissue antigens (PTAs) in medullary thymic epithelial cells (mTECs); however, AIRE has other protein partners that facilitate its activity (1, 2).

The transcriptional expression of PTAs and their protein translation is essential for the maintenance of immunological self-tolerance, which prevents the development of aggressive autoimmunity. This function is based on studies showing that mutations that provoke functional inactivation of the Aire gene in both humans and mice lead to the development of multi-organ autoimmunity (2–9).

Genes regulated by AIRE were revealed by a comparison of mTEC microarray expression profiles from wild-type and AIRE-knockout (KO) mice. These studies demonstrated that AIRE deficiency had a profound effect on the expression of many but not all PTA genes in mTECs; these genes are considered AIRE-dependent PTAs (10, 11). According to a recent study, Aire-independent PTAs are controlled by the Fez family zinc finger 2 (Fezf2) gene/protein (12, 13).

Previously, we demonstrated that slight variations in wild-type thymic Aire gene expression *in vivo* were sufficient to affect the expression of AIRE-dependent PTAs, either spontaneously as observed in the non-obese diabetic (NOD) mouse strain (14) or following induction by gene knockdown either *in vivo* in the thymuses of BALB/c strain mice or *in vitro* in a mTEC cell line (15).

AIRE is considered a non-classical transcription factor (TF) and is strongly implicated in the regulation of a large set of PTA genes in mTECs. The proteins encoded by these genes and expressed in the thymus represent all of the body tissues and organs. Due to its comprehensiveness, this ectopic type of gene expression is referred to as promiscuous gene expression (PGE) (1, 16–26).

The precise molecular mechanisms used by AIRE to regulate the transcription of thousands of genes are the subject of intense study. Various hypotheses have been proposed to explain AIRE’s mechanism of action, including direct DNA-binding in the formation of a large DNA-binding complex, the induction of elongation by recruitment of kinase positive transcription elongation factor b (pTEFb), the direct binding to histone H3 (27, 28), and the recruitment of genes to the nuclear matrix (1, 27–31). Abramson et al. (1) demonstrated that AIRE directly or indirectly interacted with 20 independent proteins involved in processes such as nuclear transport, chromatin binding/structure, transcription, and pre-mRNA processing.

Evidence indicates that AIRE activates the ectopic transcription of PTAs not only through the recognition of specific gene promoters but also by releasing blocked RNA Pol II on the chromatin (32).

Control of PGE may be more complex and may involve epigenetic mechanisms and posttranscriptional regulation involving miRNAs (5, 28, 33, 34).

Studies have revealed the expression patterns and the roles of miRNAs in the thymus. Our group demonstrated for the first time that the Aire gene regulated the expression of miRNAs in mTECs (35). Additionally, miRNAs in the thymus were associated with T cell development, posttranscriptional regulation of the Aire mRNA and PGE, maintenance of the thymic architecture, and thymic cellularity (33, 34, 36, 37).

These findings have raised interest in many questions that remain poorly understood, including the possible relationship between Aire and miRNAs in the control of central tolerance.

In this context, our group (35, 38) and the groups of Kyewski/Ucar (33, 34) independently demonstrated the existence of a link between Aire, the mRNAs encoding PTAs, and miRNAs in mTECs. In this study, we raised the hypothesis that a partial reduction in Aire expression was sufficient to imbalance this link and consequently influence PGE in mTEC cells.

To test this hypothesis, we induced transient changes in the Aire transcript levels through *in vivo* intrathymic electro-transfection of a small interfering RNA (anti-Aire siRNA), which allowed us to profile Aire-dependent miRNAs and mRNAs. The reconstruction of the miRNA–mRNA interaction networks using data from control cells or cells subjected to Aire-knockdown enabled us to demonstrate that the reduction of Aire expression was sufficient to imbalance the interaction between the mRNAs that encoded PTAs with miRNAs, thereby influencing PGE in mTECs.

## Materials and Methods

### Animals

Female BALB/c mice (5–6 weeks old), weighing 18–22 g each, were used for the experiments. The animals were housed in air-filtered isolators in temperature-controlled rooms (22°C) in the Central Animal Facility of the Ribeirão Preto Medical School, University of São Paulo, Brazil. The mice were housed under 12-h light/dark cycles and received water and food *ad libitum*.

All experimental procedures were approved by the local Ethical Committee on Animal Experimentation of the Ribeirão Preto Medical School (Permit # 1172008).

### *In Vivo* Aire Gene Knockdown

We used the TriFecta (IDT, Integrated DNA Technologies, Coralville, IA, USA) anti-Aire siRNA sequence (GGAUUCUCUUUAAG-GACUACAAUCTAGAUUGUAGUCCUUAAAGAGAAUCCUC) to knock down the Aire mRNA *in vivo* by thymic electro-transfection of BALB/c mice as previously described (15, 39). Briefly, the mice were anesthetized by intraperitoneal injection with a mixture of ketamine (Sigma-Aldrich K-003) (100 mg/kg body weight) and xylazine (Sigma-Aldrich X1251) (10 mg/kg body weight). A crocodile clip corresponding to the cathode was used to establish skin contact on the paw opposite the injection site. A 0.33-mm-diameter injection needle was connected to the crocodile clip corresponding to the anode and then introduced between the first and second ribs where the thymus was located at a 45° angle from the longitudinal axis. As previously reported (15), we observed that inserting the needle to a 5-mm depth was necessary and sufficient to reach the thymus without affecting proximal vital organs such as the heart and lungs.

After the insertion of the needle, 5 μl of sterile phosphate-buffered saline (PBS) or 5 μl of sterile PBS containing 5 μM of the Aire siRNA was slowly injected into each thymic lobe (5 μM siRNA per lobe for a total of 10 μM siRNA per thymus). An electrical current of five 20-ms pulses of 300 V was immediately applied using a standard wave electroporator (Model ECM 830, BTX^®^ Harvard Apparatus, Holliston, MA, USA).

The animals were sacrificed by CO_2_ inhalation 24 h after the electro-transfection. The thymuses were immediately removed by surgery and processed for the whole thymic stroma preparation and mTEC separation. All experiments were performed in triplicate.

This model system was previously evaluated to assess the electro-transfection delivery efficiency, Aire siRNA specificity, and tissue injury. The results showed that the thymus tissue structure was preserved within the evaluated time frame (48 h post-electro-transfection) (15).

### Medullary Thymic Epithelial Cell Isolation

The thymuses were dissected 48 h post-electro-transfection, and the thymic stroma was separated as previously described (15, 40). Briefly, the thymuses were dissected and trimmed of fat and connective tissue. The tissue fragments were gently agitated in 50 ml of RPMI 1640 medium at 4°C with a magnetic stirrer for 30 min to remove the majority of the thymocytes. The resulting thymic fragments were subsequently transferred to 10 ml of fresh RPMI 1640 medium, and the remaining thymocytes were dispersed by successive pipetting. The culture medium was changed two to three times after agitation, and the fragments were recovered by settling each time. Then, the thymic fragments were incubated in 5 ml of 0.125% (w/v) collagenase type II and 0.1% DNase I (Invitrogen, Carlsbad, CA, USA) in RPMI 1640 medium at 37°C for 15 min and gently agitated every 5 min with a 1-ml pipettor. After three to four digestions, whole stromal cells were pooled and centrifuged at 450 × *g* for 5 min and finally resuspended in 200 μl of PBS. These cells were used for mTEC isolation.

For mTEC cell isolation, we used a modified version of the Kont protocol (41). In this protocol, magnetic microbeads are used for cell isolation, and the purity is determined by flow cytometry. Briefly, whole thymic stromal cells were obtained as described above and resuspended at a density of 1 × 10^8^ cells in 98 μl of RPMI 1640 plus 2 μl of a rat anti-mouse IgG2a BP1 antibody (e-Bioscience, San Diego, CA, USA). The mixture was incubated at room temperature for 15 min. Then, the cells were mixed with magnetic microbeads coated with anti-rat IgG (MACs Miltenyi Biotec Inc., Auburn, CA, USA) to remove the cortical thymic epithelial cells (CD45^−^ Epcam^+^ BP1^+^ cTECs), which were retained in the column.

The cell fraction obtained by positive selection was incubated with a rat anti-mouse Epcam antibody and mixed anti-rat IgG magnetic microbeads. The total CD45^−^ Epcam^+^ BP1^−^ mTECs were separated on columns in a magnetic field (MACS, Miltenyi Biotec).

To determinate the phenotype of these cells, the cells were stained with a phycoerythrin (PE)-labeled anti-CD80 and a fluorescein isothiocyanate (FITC)-labeled anti-MHC class II antibody and analyzed using a FACSCalibur flow cytometer (BD Biosciences). The purity of the CD80^+^ mTECs was ≥68%, of which ≥27% were MHC class II^+^ mTEC cells (Figures S1 and S2 in Supplementary Material). These cells were used for total RNA preparation.

### Total RNA Preparation

Total RNA was extracted from the mTEC cells using the mirVana kit^®^ (Ambion, NY, USA) according to the manufacturer’s instructions. The RNA preparations were confirmed to be free of proteins or phenol by UV spectrophotometry. RNA degradation was assessed by microfluidic electrophoresis using Agilent RNA Nano 6000 chips and an Agilent 2100 Bioanalyzer (Agilent Technologies, Santa Clara, CA, USA). Only RNA samples that were free of proteins and phenol and featured an RNA integrity number (RIN) ≥9.0 were selected for quantitative reverse transcription real-time PCR (qRT-PCR) and microarray hybridization.

### Reverse Transcription-Quantitative Real-time PCR

The Aire transcript levels were assayed by reverse transcription-quantitative real-time PCR (RT-qPCR). The Primer3 web tool^1^ was used to select pairs of oligonucleotide primers spanning an intron/exon junction with an optimal melting temperature of 60°C. The Aire cDNA sequence (GenBank acc NM 009646.1) was retrieved from the NCBI GenBank database.^2^ The forward and reverse sequences were as follows: 5′ GCAACTCTGGCCTCAAAGAG 3′ and 5′ GGTCTGAATTCCGTTTCCAA 3′. AIRE transcriptional expression was quantified using a StepOne Real-Time PCR System (Applied Biosystems, USA). The Aire expression values were normalized to the expression of the housekeeping gene GAPDH (GenBank acc NM 008084.2), which is commonly used as a reference. The forward and reverse sequences for the cDNA of this gene were as follows: 5′ GGGTGTGAACCACGAGAAAT 3′ and 5′ CCTTCCACAATGCCAAAGTT 3′. All experiments were performed in triplicate. We used GraphPad Prism 5.0^3^ to run Student’s *t*-test (*p* < 0.05).

### Immunofluorescence

We followed a previously published protocol (15). Briefly, 48 h after Aire siRNA or PBS electro-transfection, the thymuses were dissected and incubated for 15 min with the 4′,6-diamino-2-phenylindole (DAPI) DNA staining solution (Molecular Probes). Then, the samples were embedded in Tissue-Tek^®^ (Sakura Finetek, USA) and cut into 5-μm sections with a histo-cryomicrotome. The sections were transferred to silane-coated slides and fixed in cold acetone for 10 min. Immunolabeling was performed using an anti-AIRE protein primary antibody (1:100 dilution of an anti-AIRE 1 goat polyclonal Sc-17986 antibody) (Santa Cruz Biotechnology) in 1% BSA, for 18 h at 4°C. A 1:200 dilution of fluorescein-conjugated rabbit anti-goat IgG (Vector Laboratories, Burlingame, CA, USA) was used as the secondary antibody. The images were analyzed with a Leica DM 6000M microscope equipped with the Leica AF6000 Deconvolution System (Leica Microsystems). All images were obtained using the same exposure time and magnification.

### Microarray Hybridizations and Data Analysis

Changes in miRNA and mRNA expression were evaluated using the Agilent one-color (Cy3 fluorochrome) microarray-based gene expression platform according to the manufacturer’s instructions.

To hybridize the mRNA to the entire functional mouse genome, a set of 4× 44 K 60-mer oligonucleotide arrays (G4122F, Agilent Technologies, Palo Alto, CA, USA) was used with 500 ng of total RNA labeled with a one-color Quick Amp labeling kit (Agilent Technologies). The raw hybridization data for the mRNAs from the control or Aire-knockdown thymuses were obtained from previous determinations performed in our laboratory.

We tested the complete miRNA set present on the 8× 15 K oligonucleotide arrays (G4470 C, miRNA V3 Microarray kit, Agilent Technologies, Palo Alto, CA, USA) for mRNA hybridization. Briefly, 100 ng of total RNA was labeled with the miRNA Complete Labeling and Hybridization Kit (Agilent Technologies). Cy3-labeled miRNA samples were hybridized for 18 h at 42°C in a rotator oven and subsequently washed. The array slides were scanned using a DNA microarray scanner (Agilent Technologies), and the hybridization signals were extracted using the Agilent Feature Extraction software (version 10.5).

The mRNA and miRNA expression profiles from independent preparations of control or Aire-knockdown mTECs were analyzed by comparing the microarray hybridizations of the respective samples.

The quantitative microarray data were normalized to the quantile and analyzed using the Agilent GeneSpring GX bioinformatics platform^4^ according to the default instructions. Hierarchical clustering of the samples was allowed based on miRNAs with fold-change ≥1.5, uncentered Pearson correlation metrics (42), and a false discovery rate (FDR) of 0.01. The similarities and dissimilarities in mRNA and miRNA expression are presented as dendrograms in which the pattern and length of the branches reflect the relatedness of the samples or mRNAs/miRNAs and as heat-maps.

A complete file including all of the miRNA and mRNA oligo probes present in the microarrays used in this study with the experimental conditions is available online in the EMBL-EBI ArrayExpress public database^5^ under accession numbers E-MEXP 3665 (for miRNAs) and E-MEXP-3343 (for mRNAs).

### Reconstruction of the miRNA–mRNA Posttranscriptional Interaction Networks

We used the GenMiR^++^ algorithm, which is available online at http://www.psi.toronto.edu/genmir/, to reconstruct the miRNA–mRNA interaction networks. This tool uses the respective miRNA and mRNA normalized expression values (Tables S1–S4 in Supplementary Material) from the same cell sample to identify candidate miRNA–mRNA target pairs that are best supported and constructs a database of a potential set of mRNA targets for each identified miRNA (43).

### Validation of miRNA–mRNA Interactions

#### Determination of the Hybridization Minimum Free Energy

All selected miRNA–mRNA interactions in the target prediction generated by the GenMiR^++^ algorithm were initially validated using the RNAhybrid algorithm available at http://bibiserv.techfak.uni-bielefeld.de/rnahybrid (44, 45).

This method involves a dynamic programing algorithm that calculates the most favorable hybridization between the seed region of a miRNA and the 3′ UTRs of the mRNA targets. The minimum free energy (MFE) is calculated based on a thermodynamic state that postulates that an RNA duplex is more stable and thermodynamically stronger when the free energy is low (46).

Interaction networks were designed considering the thermodynamically validated miRNA–mRNA pairs using the Cytoscape v 2.1 graphical program, which is available online at http://www.cytoscape.org.

#### Luciferase Reporter Gene Assay

We used the luciferase reporter gene assay (LRGA) to validate whether the selected miRNA–mRNA interactions that featured the lowest hybridization MFE could occur within the cellular milieu.

This approach was previously described by our group (38, 47, 48). Briefly, double-stranded complementary oligonucleotide pairs containing a portion of the Hmga2 or Prpsap1 3′ UTR with the predicted miRNA binding sites were synthetized by Sigma-Aldrich (St. Louis, MO, USA).

These sequences were cloned into *E. coli* TG1 using the pmirGLO Vector (Promega Corporation, USA) between the *Xho*I/*Xba*I restriction sites, resulting in the miRNA target region in the correct 5′ to 3′ orientation immediately 3′ downstream of the luciferase gene. For selected targets, we introduced point mutations into the 7-nt seed-binding sequence. These constructs (named “pMIR-Hmga2” and “pMIR-Prpsap1” for the sequences) were selected by colony PCR using the following primer pairs: forward 5′ AGGTTACAACCGCCAAGAAG 3′ and reverse 5′ CAGCCAACTCAGCTTCCTTT 3′; these primers flank the vector polycloning site.

For the LRGA, 0.2 μg of each pmirGLO construct was transfected together with 1.6 pmol of miR-let-7a, miR-378, or a scrambled miRNA (Thermo Scientific Dharmacon, Waltham, MA, USA) into HEK-293T cells (6 × 10^4^ cells/well) in a 96-well plate. The transfections were performed using the Attractene Transfection Reagent (Qiagen, Hilden, Germany) according to the manufacturer’s instructions. The transfected cells were incubated at 37°C in a 5% CO_2_ incubator. Twenty-four hours after transfection, the cells were lysed in passive lysis buffer. Then, the firefly and Renilla luciferase activities were measured in a BioTek Synergy 2 luminometer (BioTek Instruments Inc., Winooski, VT, USA) according to the manufacturer’s instructions. The statistical analysis of the results is presented as the SEM. The differences were evaluated by one-way ANOVA (*p* < 0.05) with Bonferroni’s multiple comparison test. Our laboratory is certified by CTNBIO, Brasília, Brazil, for recombinant DNA manipulations under biosafety level 1 (permit # 040/1996).

### Determination of Promiscuous Gene Expression

mRNA targets that established interactions with miRNAs were categorized in terms of their encoded protein antigens that represented tissue/organs. This approach enabled us to characterize PGE based on combined information from the BioGPS public database.^6^ This database shows gene expression in more than 60 mouse tissue/organs assessed by gene array analysis.

In this study, genes that were ectopically expressed in the thymus were selected based to their relative expression levels in different tissues/organs. Only genes with expression values greater than the median in relation to all other tissues were selected.

## Results

### *In Vivo* Aire Gene Knockdown in mTEC Cells Evaluated at the mRNA Level

**Figure 1:**
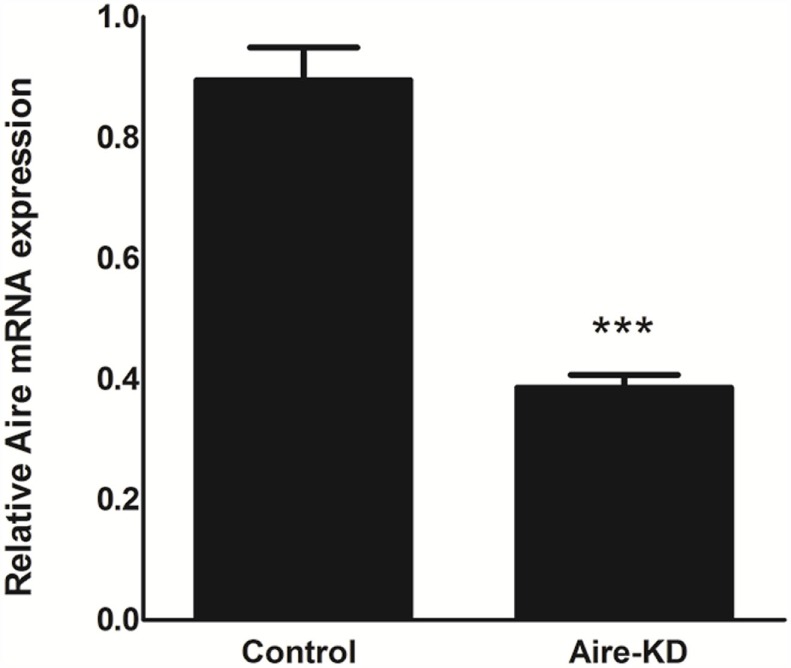
**Aire mRNA expression levels were analyzed by RT-qPCR in mTEC cells 48 h after electro-transfection with anti-Aire siRNA**. Aire expression was decreased by 57% in Aire-knockdown when compared to control mTECs. Student’s *t*-test, *p* < 0.05, KD, knockdown.

The RT-qPCR analysis showed that the Aire mRNA levels were downregulated by approximately 57% 48 h after *in vivo* thymic electro-transfection of the anti-Aire siRNA (Figure 1).

### *In Vivo* Aire Gene Knockdown in mTEC Cells Evaluated at the Protein Level

**Figure 2:**
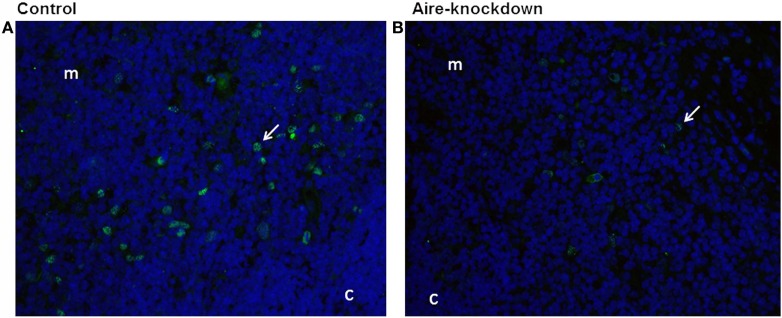
**Immunofluorescence for Aire protein 48 h after anti-Aire siRNA electro-transfection**. **(A)** Thymus electro-transfected with PBS buffer (control) or **(B)** electro-transfected with anti-Aire siRNA. Nuclei of cells were stained with DAPI (blue) and for AIRE protein in green by using goat anti-Aire IgG primary antibody and fluorescein-conjugated rabbit anti-goat IgG secondary antibody. Comparing to the control thymus, immunofluorescence signal for AIRE protein was strikingly reduced in the nuclei of cells of Aire-knockdown thymus. Magnification 40×, c, cortex; m, medulla.

After demonstrating by RT-qPCR that the *in vivo* anti-Aire siRNA electro-transfection was an effective method to reduce Aire mRNA expression in the thymus, immunofluorescence microscopy was used to detect the AIRE protein in thymus tissue sections under Aire-knockdown conditions. Figure 2 shows the detection of this protein (green fluorescence) exclusively in cells of the thymic medullary compartment. The thymus under Aire-knockdown conditions featured a reduction in the number of Aire^+^ cells and in the intensity of the fluorescence signal, which reflected a reduction in AIRE protein expression. Moreover, immunoblotting was used to measure the AIRE protein levels under the Aire-knockdown conditions. Figure S3 in Supplementary Material demonstrates the detection of the 55 kDa AIRE protein in the control thymus and an approximately 50% reduction of this protein in the Aire-knockdown thymus.

These results revealed that *in vivo* intrathymic electro-transfection of the anti-Aire siRNA was an effective procedure to reduce AIRE protein levels at the examined time point.

### Differentially Expressed miRNAs under AIRE Gene Knockdown Conditions

**Figure 3:**
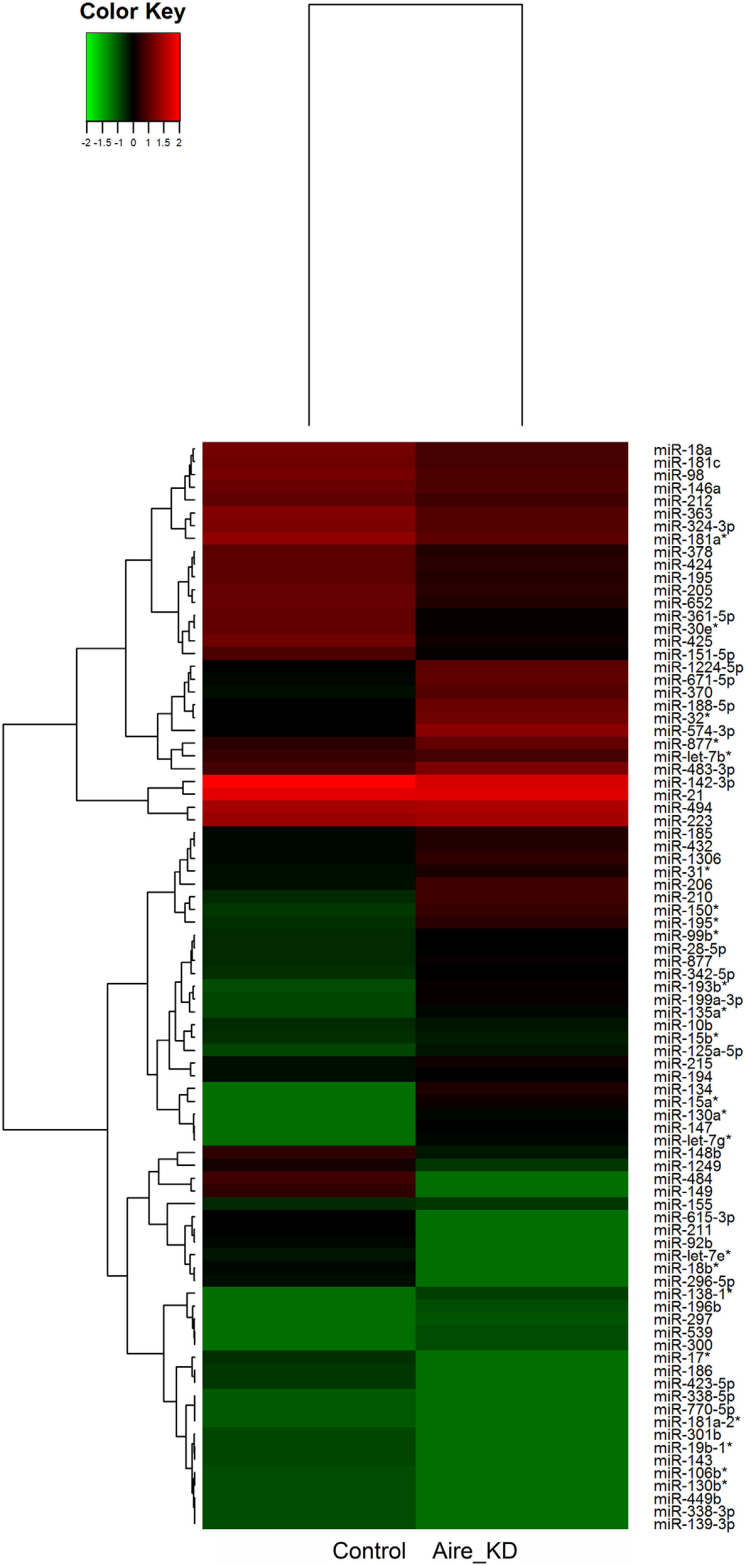
**Hierarchical clustering and color heat-map of miRNAs differentially expressed comparing control to Aire-knockdown mTEC cells**. The dendrogram and heat-map were obtained using cluster and treeview algorithms through Agilent GeneSpring platform. Heat-map color legend: red, upregulation, green, downregulation, black, unmodulated (Pearson correlation metrics, fold-change ≥1.5, FDR 0.01). KD, knockdown.

We identified 87 differentially expressed miRNAs (*p* > 0.05, fold-change ≥1.5) after comparing the control with the Aire-knockdown mTEC cells. Hierarchical clustering of the data allowed the identification of clusters of downregulated (repressed) and upregulated (induced) miRNAs (Figure 3), of which 44 were upregulated and 43 were downregulated. These data strongly suggest that these miRNAs are controlled by Aire.

### Differentially Expressed mRNAs under Aire Gene Knockdown Conditions

**Figure 4:**
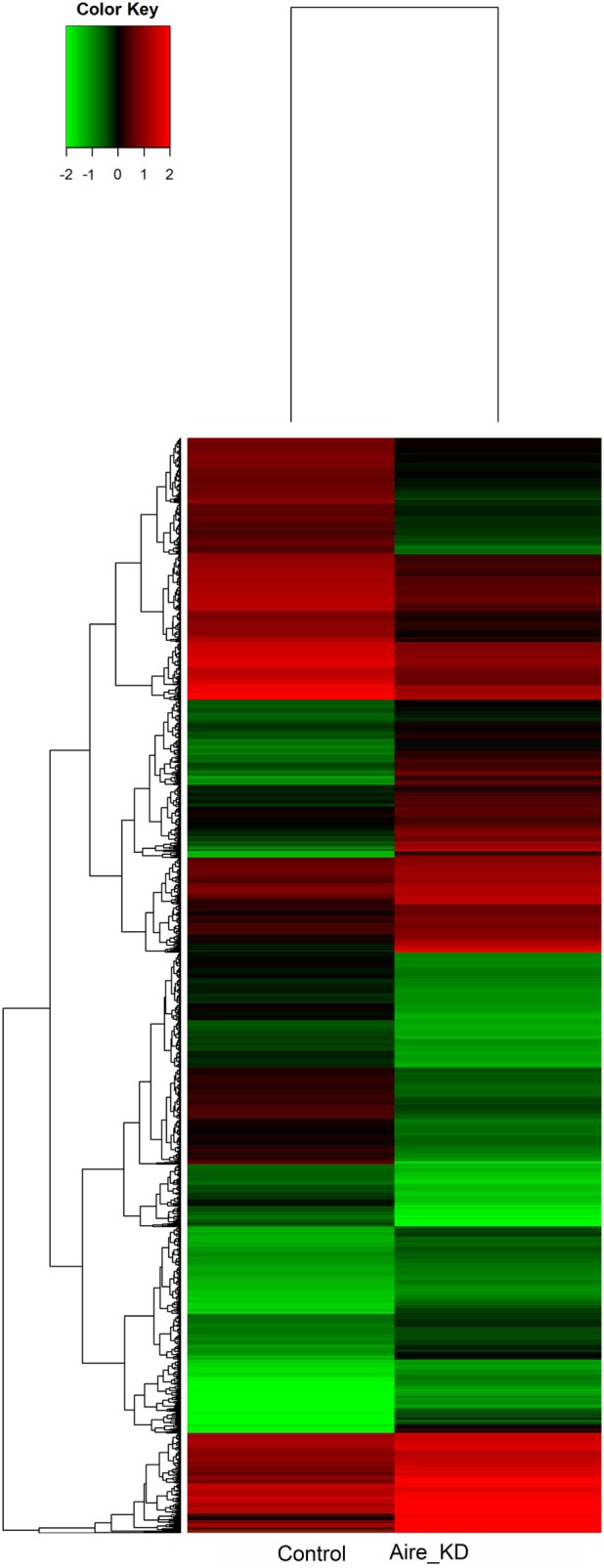
**Hierarchical clustering and color heat-map of mRNAs differentially expressed comparing control to Aire-knockdown mTEC cells**. The dendrogram and heat-map were obtained using cluster and treeview algorithms through Agilent GeneSpring platform. Heat-map color legend: red, upregulation, green, downregulation, black, unmodulated (Pearson correlation metrics, fold-change ≥1.5, FDR 0.01). KD, knockdown.

The microarray analysis comparing the control with the Aire-knockdown mTEC cells presented a control/test ratio of ~1.0 (Pearson correlation) and a fold-change ≥1.5. The analysis revealed that 4,558 mRNAs were differentially expressed under Aire-knockdown conditions. Of these genes, 2,326 were downregulated (repressed) and 2,232 were upregulated (induced) (Figure 4).

### Interaction Networking for miRNA–mRNA Target Prediction

**Figure 5:**
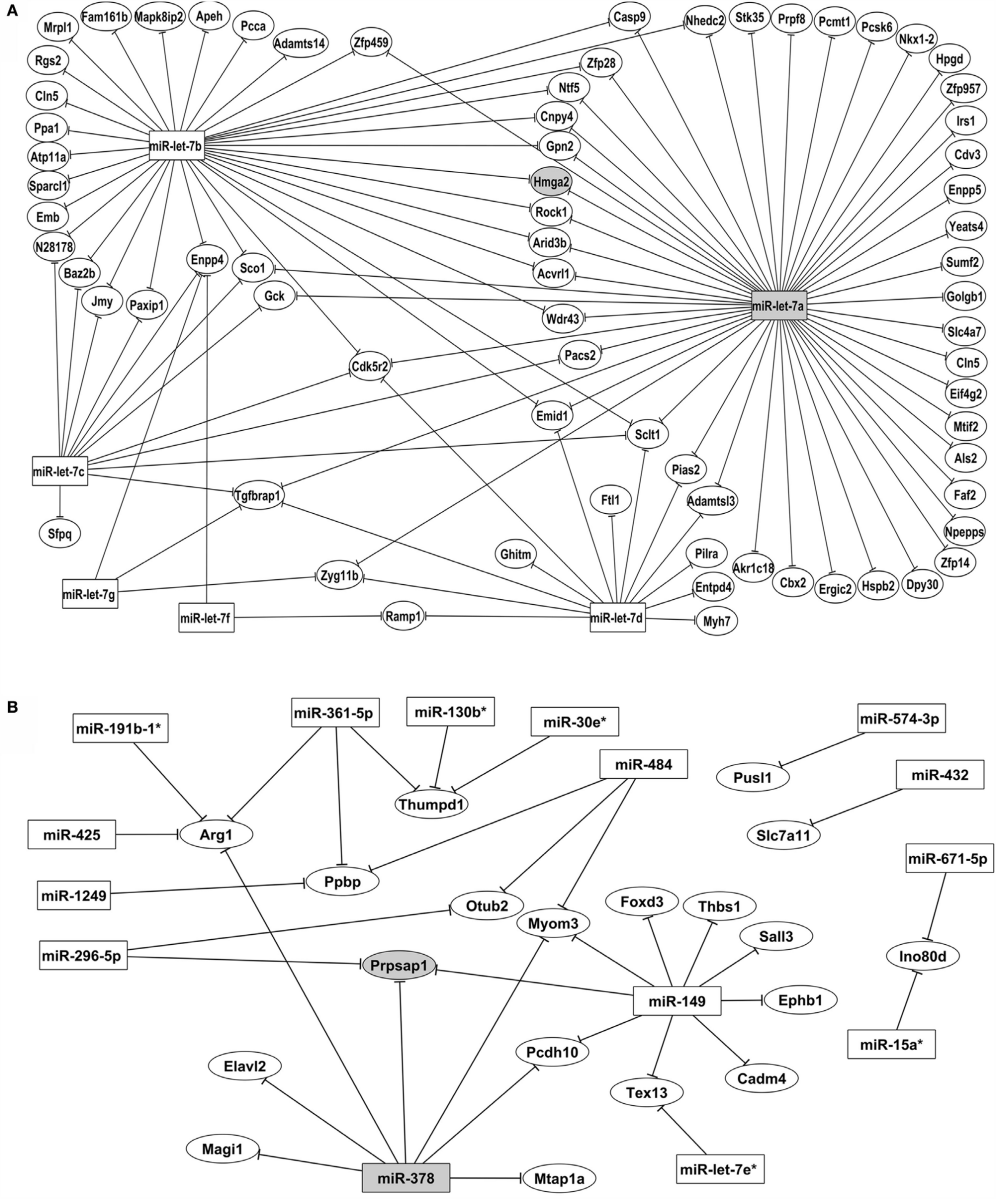
**Posttranscriptional miRNA–mRNA interaction network based on microarray expression data**. The network of control mTEC cells **(A)** shows the participation of six miRNAs of let-7 family that interact with PTA mRNAs targets. The highlighted interaction (let-7a–Hmga2 mRNA) was selected for further validations. The network of Aire-knockdown mTEC cells **(B)** shows the participation of 15 miRNAs from different families that interact with a restricted group of PTA mRNA targets. The highlighted interaction (miR-378–Prpsap1 mRNA) was selected for further validations. Interaction networks were reconstructed by using the GenMir^++^ algorithm, which consider the respective modulation of miRNAs (upregulated) and of mRNA targets (downregulated).

The microarray-normalized data were used as inputs for the GenMir^++^ algorithm to reconstruct two miRNA–mRNA interaction networks (Figures 5A,B). The thermodynamic hybridization stabilities of the selected miRNA–mRNA pairs were reanalyzed using the RNAhybrid algorithm, which calculated the MFE of annealing. We selected only miRNA–mRNA pairs with an MFE ≤−20 kcal/mol for the subsequent experiments.

**Figure 6:**
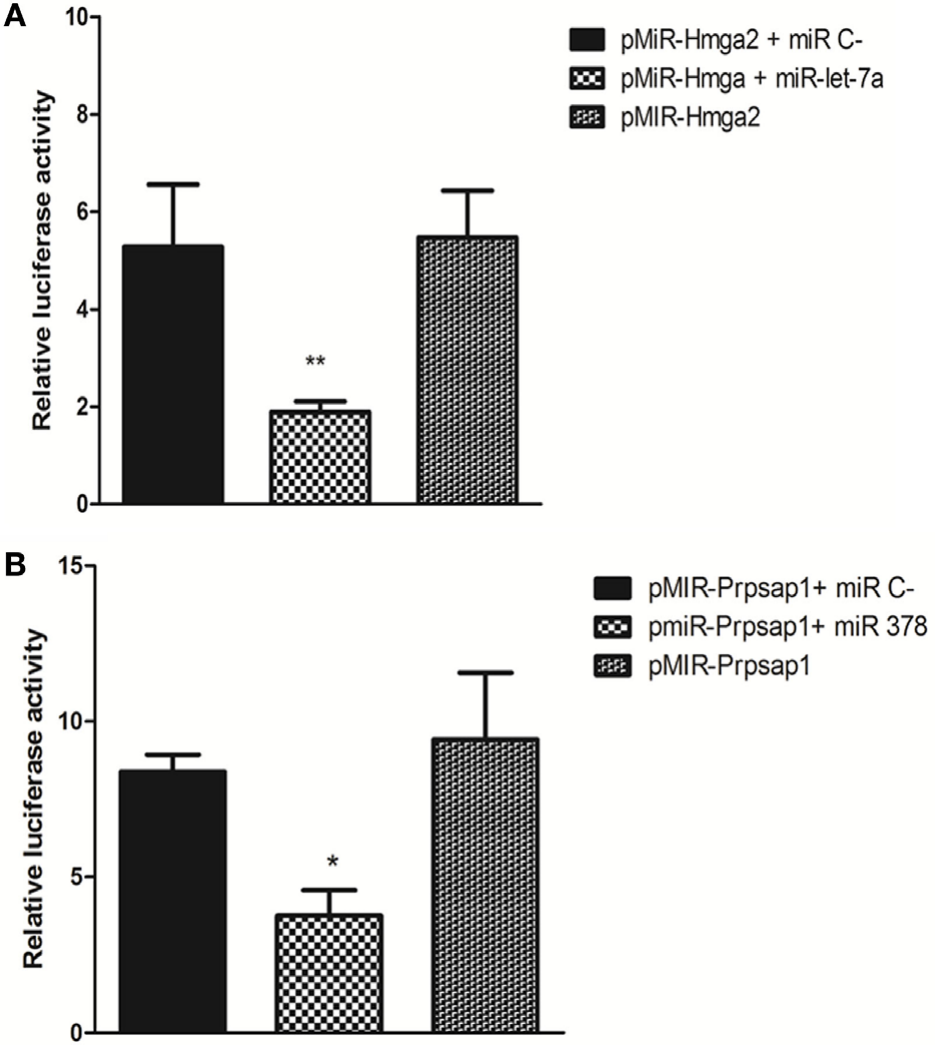
**Luciferase reporter gene assay**. pMIR-Hmga2 or pMIR-Hmga2 (M) 3′ UTR luciferase plasmid were co-transfected with control irrelevant miR or with miR-let-7a mimic **(A)**, pMIR-Prpsap1 or pMIR-Prpsap (m) plasmid were co-transfected with control irrelevant miR or with miR-378 mimic **(B)** into HEK-293T cells. Luciferase activity was evaluated at 24 h post-transfections. These data are presented as means and SEM. The differences were evaluated by a one-way ANOVA followed by Bonferroni’s test. *N* = 5; means ± SEM (*p* < 0.001).

The interaction network depicted in Figure 6A refers to the control mTECs and indicates that only six members of the let-7 miRNA family (miR-let-7a, miR-let-7b, miR-let-7c, miR-let-7d, miR-let-7f, and miR-let-7g) interacted with their respective mRNA targets.

Figure 6B depicts the interactions of AIRE-knockdown mTECs. Here, 15 miRNAs (miR-let-7e*, miR-15a*, miR-19b-1*, miR-30e*, miR-130b*, miR-149, miR-296-5p, miR-362-5p, miR-378, miR-425, miR-432, miR-484, miR-574-3p, miR-671-5p, and miR-1249) established interactions with 19 mRNAs.

### Validation of miRNA–mRNA Interactions Using the Luciferase Reporter Gene Assay

We used the LRGA to validate the occurrence of two miRNA–mRNA interactions within the cellular milieu by focusing on the Hmga2 and Prpsap1 mRNA targets. We confirmed that miR-let-7a interacted with the Hmga2 3′ UTR and that miR-378 interacted with the Prpsap1 3′ UTR. The aforementioned miRNAs hybridized with their respective 3′ UTR sequences containing their predicted binding sites (Figures 6A,B).

### Tissues/Organs Representation by Promiscuous Gene Expression

**Figure 7:**
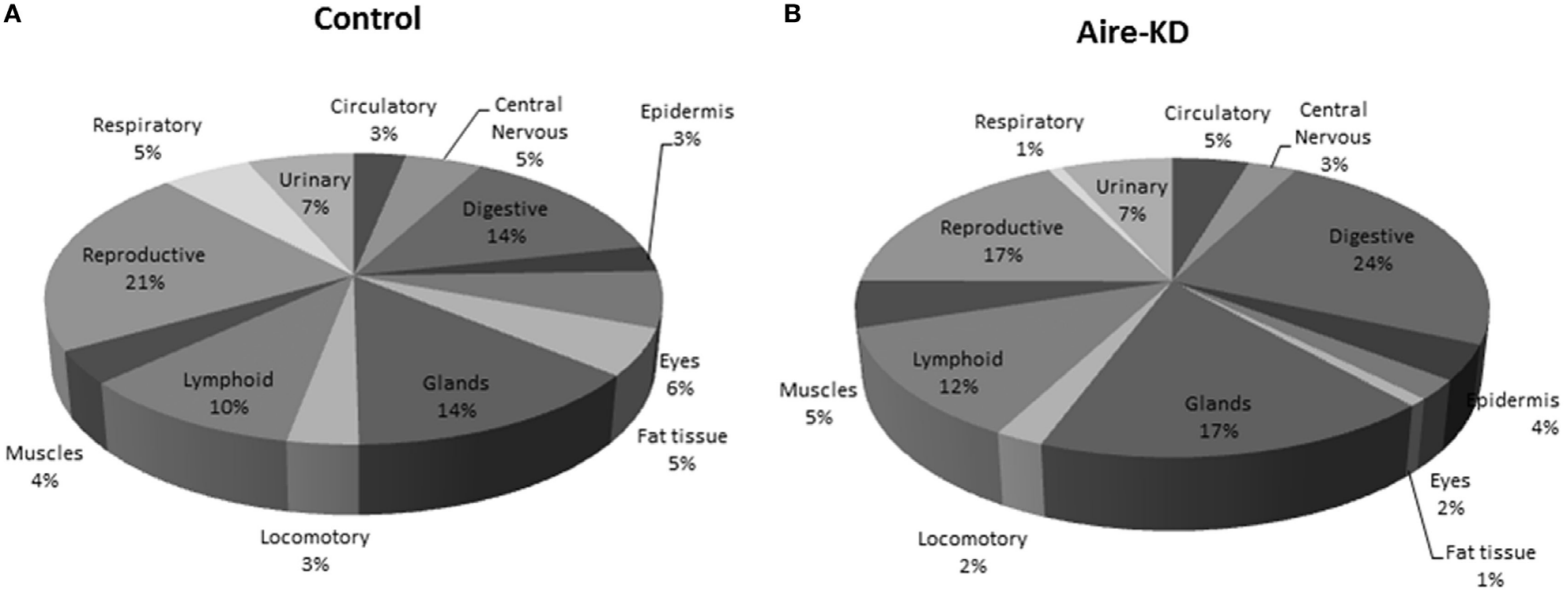
**Representation of tissue/organ systems by transcriptional gene expression (PGE) considering those mRNAs participating on miRNA–mRNA interaction networks of control mTEC cells (A) or Aire-knockdown mTEC cells (B)**. The mRNAs were subgrouped into 13 anatomic functional body systems according to their respective protein products. Under Aire-knockdown, mTEC cells change the profile of self-representation.

The 72 mRNA targets present in the miRNA–mRNA control interaction network and the 19 mRNAs present in the Aire-knockdown interaction network were assigned to 13 anatomic functional body systems as follows: lymphoid, reproductive, respiratory, urinary, circulatory, locomotor, central nervous, digestive and muscles, epidermis, eyes, fat tissue, and glands (Figures 7A,B).

## Discussion

In this work, we showed that the Aire expression level in mTEC cells was crucial. Aire downregulation caused by *in vivo* electro-transfection with an anti-Aire siRNA in the mouse thymus altered the miRNA and mRNA expression levels. Because the intracellular amounts of these RNAs are one important factor that interferes with their interactions, one consequence of this knockdown was changes in the miRNA–mRNA interaction networks (i.e., the posttranscriptional control of mRNAs encoding PTAs in the mTECs).

AIRE expression is a characteristic of mature mTECs (49–51). This protein has the ability to direct the expression of hundreds or even thousands of peripheral tissue antigens (PTAs) in a process known as PGE that may be essential for the induction of self-tolerance (19).

Two independent strains of AIRE-deficient mice have been established. These mouse strains develop multi-organ autoimmunity, inflammatory infiltrates, and serum autoantibodies (9, 52). The overall pattern of multi-organ autoimmunity is consistent between the AIRE-deficient mouse strains and humans, especially individuals with a specific syndrome called autoimmune polyendocrinopathy-candidiasis-ectodermal dystrophy (APECED), which is a monogenic autoimmune disease caused by mutations in the Aire gene sequence (8, 53). Based on these observations, the Aire gene was strongly associated as a controller of autoimmunity, which served as the basis for the gene name.

Aire has been proposed to function as a non-classical TF based on an understanding of the mechanisms of classical TF actions. Classical TFs directly operate by interacting with specific promoter DNA sequences and controlling the activity of only a few genes. However, the Aire/AIRE gene/protein controls hundreds or even thousands of PTAs. Thus, questions remain concerning how this gene can have many DNA-binding sites in mTEC cells.

Evidence has revealed that the AIRE protein acts indirectly. AIRE associates with RNA Pol II when it is stalled close to the promoter regions of target genes along the chromatin. Thus, AIRE is involved in the elongation of PTA gene transcripts (32). This observation greatly clarified the nature of AIRE’s wide range of actions and consequently explained the mechanism by which AIRE controlled PGE.

Moreover, AIRE has been suggested to regulate the transcription of PTA genes through the mediation of more restricted TF sets or *via* miRNAs (16). Studies have suggested that PGE may be regulated by other mechanisms in addition to AIRE, such as epigenetics and/or posttranscriptional control (33–37). Recently, PGE regulation of the controller function on the set of Aire-independent PTA genes was assigned to the FEZ family zinc finger protein 2 (Fezf2) gene (12, 13).

Based on previous observations made by our group and others that (1) slight variations in Aire expression imbalanced PGE in the thymus of NOD mice (14), (2) in addition to PTAs, Aire also controlled miRNAs in mTECs (35), and (3) miRNAs played roles in the control of PGE (33–35, 37), in this study we hypothesized that the influence of Aire in mTEC cells occurred quantitatively (i.e., the expression levels were important and impacted both mRNAs and miRNAs).

Accordingly, we hypothesized that a partial reduction in Aire expression could be sufficient to perturb mRNA–miRNA interactions in mTECs.

This demonstration may indicate that not only mutations in the Aire gene sequence but also variations in its expression levels are important for the transcriptional and posttranscriptional control of PGE.

To test this hypothesis, we used a model system previously used by our group that consisted of the *in vivo* downregulation of Aire by electroporation (electro-transfection) with an anti-Aire siRNA introduced directly into the mouse thymus (15).

As previously discussed (15), Aire-KO mice represent an adequate model to evaluate the effect of complete annulment of Aire because the gene segment is disrupted in these animals and its expression is completely abolished. However, the KO animals were not a suitable model to test whether slight changes in Aire expression exerted an effect on downstream gene expression of mTECs.

In contrast, naked siRNAs do not integrate into the genome and consequently do not disrupt DNA sequences that may be important for the cellular transcription machinery. Accordingly, in this study we used siRNA-mediated Aire gene knockdown, which preserved the gene in the genome but caused transient and partial degradation of the target mRNA.

The sets of modulated (upregulated or downregulated) mRNA or miRNAs in isolated mTEC cells were identified using the microarray technique; these data were used to trace gene expression signatures and reconstruct posttranscriptional interaction networks for miRNA–mRNA target prediction.

The *in vivo* knockdown of Aire in mTEC cells from BALB/c mice was effective after 48 h post-electro-transfection as demonstrated by the mRNA level detected by qRT-PCR (Figure 1) and the protein level detected by immunofluorescence (Figure 2) and western blotting (immunoblotting) (Figure S3 in Supplementary Material). As we previously demonstrated, although this type of perturbation in Aire was partial and transient because it was caused by siRNA, the knockdown could be sufficient to affect large number of PTAs (15). Therefore, this model system appears to be appropriate to test our hypothesis.

The transcriptional profiling comparing the control with the Aire-knockdown mTECs revealed that 87 miRNAs and 4,558 mRNAs were differentially expressed (i.e., upregulated or downregulated) (Figures 3 and 4). The modulated miRNAs were considered Aire-dependent (Figure 3). This observation was consistent with our previous observations using an *in vitro* model system of an mTEC cell line in which Aire downregulation also provoked the upregulation or downregulation of miRNAs (35). This finding is consistent with previous observations that TFs including Aire can activate or even suppress miRNA expression and therefore can serve as upstream regulators of these molecules (11, 54). As an upstream regulator, our results suggest that Aire’s mode of action on the posttranscriptional regulation of PTAs is indirect but effective.

From the set of the 87 Aire-dependent miRNAs identified in this study, we identified 6 miRNAs (miR-10, miR-30e*, miR142-3p, miR-425, miR-338-3p, and miR-297) that were shared with the set of Aire-dependent miRNAs identified by *in vitro* Aire-knockdown of an mTEC cell line (35) and 4 miRNAs (miR-206, miR-10b, miR-181c, and miR-363) whose expression was previously detected in TECs. For instance, miR-206 was upregulated in cTECs, mTEC^high^, and mTEC^low^; miR-10b was downregulated in cTECs but upregulated in mTEC^high^ and mTEC^low^; miR-181c was downregulated in cTECs and mTEC^high^ but upregulated in mTEC^low^; and miR-363 was downregulated in cTECs, mTEC^low^, and mTEC^high^ (33).

Aire is involved in the maturation of mTEC cells (55), which suggests that an association may exist between Aire and at least these four miRNAs during the mTEC differentiation process. This possibility represents an unanswered question in this study that is open for further study.

Moreover, miRNA expression in the thymic epithelium has been described as essential for the maintenance of central tolerance. The lack of Dicer or DGCR8 causes premature thymic involution and diminished T-cell output and contributes to the development of autoimmunity (33, 36, 56, 57).

Additionally, evidence has begun to accumulate demonstrating that miRNAs may be involved in Aire and PGE regulation (33–35, 37).

Therefore, to better understand the role of Aire and miRNAs in the regulation of PGE, we reconstructed miRNA–mRNA interaction networks. For this purpose, we choose the GenMir^++^ program (43), which establishes network interactions based on Bayesian statistics of opposing miRNA and mRNA expression levels from actual microarray data.

Our results allowed us to determine which miRNAs controlled PTAs in the mTECs and whether the Aire expression levels could affect these interactions. As expected, we generally observed that one given miRNA interacted with several mRNA targets and that one mRNA could interact with several miRNAs at once. The miRNA–mRNA interactions changed under Aire-knockdown with no interaction in common with the network originating from the control mTECs (Figures 5A,B).

One notable result from this study is the participation of only miRNAs from the let-7 family (let-7a to let-7g), which interact with a large number of PTA mRNAs in the network of control mTEC cells (Figure 6A). These miRNAs belong to the highly conserved let-7 miRNA family, which harbors 12 genes encoding 9 different miRNAs (let-7a to let-7i). The let-7b* and let-7e miRNAs were differentially expressed compared with the expression patterns in mTEC cells isolated from newborn or adult pre-diabetic NOD mice. However, only let-7b* interacted with the PTA mRNAs (38).

Among the miRNAs participating in the control mTEC miRNA–mRNA interaction network, we highlighted miR-let-7a because it interacted with the largest number of mRNA targets (Figure 4A).

Interestingly, after Aire-knockdown, a large number of miRNAs interacted with their respective targets, and miR-149 exhibited an increased number of interactions with the PTA mRNAs. This result is the first evidence for miR-149 participation in the thymus (Figure 4B).

Although most of the miRNA–mRNA interactions observed in this study were new, any previously described Aire-dependent PTA interacted with the miRNAs, showing that they were refractory. In fact, previous findings from our group showed that Aire-dependent PTAs exhibited pronounced refractoriness with miRNAs in mTECs isolated from BALB/c or NOD mice (38). Therefore, the 3′ UTR portion of these PTA mRNAs may potentially be altered, thereby conferring a refractoriness to the miRNA interaction.

We choose the miR-let-7a–Hmga2 mRNA and miR-378–Prpsap1 mRNA interactions for validation through LGRA because these mRNAs encoded PTAs and therefore could be adequately used as references (Figures 6A,B). Additionally, these two interactions were predicted in the MiRWalk database,^7^ which integrated other databases, including Diana Tools,^8^ miRanda,^9^ RNAhybrid,^10^ PicTar,^11^ Pita,^12^ RNA22,^13^ and TargetScan.^14^

Most studies on the action of miRNAs are based on bioinformatics target predictions, which make the results somewhat elusive. To try to minimize this type of uncertainty, in this study we reconstructed the interaction networks only with the miRNA–mRNA pairs featuring thermodynamically stable hybridizations, followed by validation by the LRGA.

Finally, we reinterpreted these data to evaluate possible immunological meaning. The mRNAs encoding PTAs represent the mediator elements of self-representation in the thymus and thus PGE should be assessed. We observed that the PGE comprehensiveness changed after Aire-knockdown (Figures 7A,B).

The representation of the different organs/tissues by mTECs through PTA mRNA expression is affected even when the Aire gene is partially downregulated. This finding clearly demonstrates the importance of the maintenance of Aire expression levels for the expression of PTAs and consequently self-representation in the thymus.

Although these aspects were not tested in this work, we speculated that the imbalance in PTA expression in mTECs might perturb negative selection and consequently the T cell repertoire by populating the periphery with autoreactive clones, which could attack tissues/organs expressing the correspondent PTAs as seen in APECED syndrome (8, 53).

Indeed, in a recent study we demonstrated that variations in Aire expression in a murine mTEC cell line *in vitro* were sufficient to hinder the nuclear localization of the AIRE protein, the expression of adhesion molecule genes, and the adhesion between mTECs and thymocytes, which is a crucial step for the occurrence of negative selection (58).

According to these results, we conclude that the Aire expression levels correlate with the posttranscriptional control of PGE. The absence of Aire, even partially, changes the posttranscriptional regulation of PTA mRNAs, thereby confirming the existence of a link between Aire and miRNAs in the control of PGE in mTEC cells as previously considered by Ucar and Rattay (34) and by our group (37).

## Author Contributions

EO and GP conceived and designed the study and wrote the manuscript. EO, CM, AF, and PD conducted the experimental work. EO and CC did the bioinformatics analyses. ES-H and ED designed the study.

## Conflict of Interest Statement

The authors declare that the research was conducted in the absence of any commercial or financial relationships that could be construed as a potential conflict of interest.

## Footnotes

^1^http://frodo.wi.mit.edu/primer3/.

^2^http://www.ncbi.nlm.nih.gov/sites/entrez?db=nuccore&itool=toolbar.

^3^http://graphpad.com/scientific-software/prism/.

^4^www.agilent.com/chem/genespring.

^5^http://www.ebi.ac.uk/arrayexpress/.

^6^http://biogps.org/#goto=welcome.

^7^http://www.umm.uni-heidelberg.de/apps/zmf/mirwalk/micrornapredictedtarget.html.

^8^http://diana.imis.athena-innovation.gr/DianaTools/index.php.

^9^http://www.microrna.org/microrna/getGeneForm.do.

^10^http://bibiserv.techfak.uni-bielefeld.de/rnahybrid.

^11^http://pictar.mdc-berlin.de/.

^12^http://genie.weizmann.ac.il/pubs/mir07/mir07_data.html.

^13^https://cm.jefferson.edu/rna22v2/.

^14^http://www.targetscan.org/.

## Supplementary Material

Figure S1 **FACS analysis for CD80^+^ mTEC cells as isolated from thymus by Miltenyi Biotec magnetic beads**.

Figure S2 **FACS analysis for MHC-II^+^ mTEC cells as isolated from thymus by Miltenyi Biotec magnetic beads**.

Figure S3 **Immunoblotting quantification for AIRE protein in control or Aire-knockdown thymus**. Densitometry analysis of immunoblotting shows 50% reduction of AIRE protein expression after Aire-knockdown when compared to the control. GAPDH protein signal was used to normalize loading differences between lanes. Student’s *t-*test (*p* < 0.05), KD, knockdown. To measure the AIRE protein levels, PBS (control) or Aire siRNA electro-transfected (Aire knockdown) thymus tissue was excised 48 h after electro-transfection (*n* = 3 animals per group) and analyzed by immunoblotting (western blotting). The thymic stroma was homogenized in extraction buffer plus a protease inhibitor cocktail (Sigma-Aldrich, St. Louis, MO, USA). The total thymic stromal proteins (20 μg protein/lane) were resolved on by 12% sodium dodecyl sulfate polyacrylamide gel electrophoresis (SDS-PAGE) and electro-transferred (electro-blotting) to a polyvinylidene difluoride (PVDF) membrane (Amersham Hybond P 0.2, GE Healthcare Life Sciences). The membranes were blocked with 5% bovine serum albumin in 1× Tris-buffered saline (pH 7.9) with 0.05% Tween 20 (BSA/TBST) for 3 h. The membranes were incubated overnight at 4°C with 1:500 dilution of an anti-AIRE 1 (D-17): Sc-17986 goat polyclonal primary antibody (Santa Cruz Biotechnology Inc., Santa Cruz, CA, USA), followed by incubation with 1:10,000 dilution of a horseradish peroxidase (HRP)-conjugated donkey anti-goat IgG Sc-2020 secondary antibody (Santa Cruz Biotechnology) for 1 h at room temperature. To control for lane loading, the membranes were probed with a goat anti-GAPDH antibody (Cell Signaling, Danvers, MA, USA; 1:10,000 dilution) after washing with stripping buffer. The membranes were developed using the peroxidase substrate for enhanced chemiluminescence (ECL) (Millipore, Billerica, MA, USA). Gel documentation and signal quantification were performed using bio-imaging analysis on a molecular imager apparatus (ChemiDoc XRS System, Bio-Rad, Richmond, CA, USA). The images were analyzed using the gel analysis function of the Image J program (available at http://rsb.info.nih.gov/ij/download.html). Analysis of the quantitative numerical value for each protein band, which was proportional to the integrated density value (IDV) of the band, corresponded to arbitrary units (AU). All experiments were performed in triplicate. We used GraphPad Prism 5.0 to run Student’s *t-*test (*p* < 0.05).
