# Supplementary material for: Aire Downregulation Is Associated with Changes in the Posttranscriptional Control of Peripheral Tissue Antigens in Medullary Thymic Epithelial Cells
Source: Front Immunol. 2016 Nov 23;7:526. doi: 10.3389/fimmu.2016.00526 (PMC5120147; doi:10.3389/fimmu.2016.00526)
Supplement: Supplementary file 1 [file table_1.docx]

Supplemental Table 1. Normalized expression values of miRNAs from control mTECs

| **miRNAs** | **Normalized expression values** |
| --- | --- |
| miR-let-7a | 0.31930256 |
| miR-let-7b | 0.24759579 |
| miR-let-7c | 0.13674927 |
| miR-let-7d | 0.28281593 |
| miR-let-7f | 0.12547588 |
| miR-let-7g | 0.19238758 |
