# Supplementary material for: Aire Downregulation Is Associated with Changes in the Posttranscriptional Control of Peripheral Tissue Antigens in Medullary Thymic Epithelial Cells
Source: Front Immunol. 2016 Nov 23;7:526. doi: 10.3389/fimmu.2016.00526 (PMC5120147; doi:10.3389/fimmu.2016.00526)
Supplement: Supplementary file 2 [file table_2.docx]

Supplemental Table 2. Normalized expression values of mRNAs from control mTECs

| **mRNA** | **Normalized expression values** |
| --- | --- |
| Hmga2 | -0.29964542 |
| Wdr43 | -0.03843403 |
| Rock1 | -0.22825718 |
| zfp459 | -0.35576153 |
| ntf5 | -0.30248356 |
| zfp28 | 0.12398243 |
| casp9 | -0.3287711 |
| nhedc2 | -0.35326767 |
| Gpn2 | -0.33574057 |
| adamts14 | -0.34993172 |
| Mapk8ip2 | -0.34993172 |
|  |  |
| cln5 | -0.3010559 |
| Pcca | -0.2965002 |
| Sparcl | -0.05242443 |
| Atp11a | -0.0704031 |
| Mrpl1 | -0.1592207 |
| Fam161b | -0.3309517 |
| Apeh | 0.037129402 |
| Baz2b | 0.013388634 |
| Jmy | 0.20100212 |
| N28178 | -0.3790598 |
| Paxip1 | -0.38059044 |
| sfpq | -0.3380723 |
| Enpp4  Supplemental Table 2. Continued | -0.045456886 |
| Ramp1  Tgfbrap1 | -0.38102055  -0.3801012 |
| Sco1 | 0.19119835 |
| Cdk5r2 | -0.36125565 |
| Zyg11b | -0.38043642 |
| Gck | -0.3593893 |
| Pacs2 | 0.013601303 |
| Ghitm | -0.0055732727 |
| Ftl1 | -0.36408234 |
| Entpd4 | -0.35526562 |
| myh7 | 0.07443237 |
| Pilra | -0.380239 |
| Arid3b | -0.3164549 |
| Acvrl1 | -0.2982969 |
| Cnpy4 | -0.062161446 |
| Emid1 | -0.32650375 |
| Sclt1 | -0.33994198 |
| Pias2 | -0.011992931 |
| adamtsl3 | -0.351326 |
| cbx2 | -0.022515297 |
| stk35 | -0.27560997 |
| slc4a7 | -0.27466297 |
| pcsk6 | -0.29030943 |
| Npepps | -0.12613487 |
| Nkx1-2 | -0.28939533 |
| Mtif2 | -0.18529844 |
| irs1 | -0.2707939 |
| Hspb2 | -0.29042387 |
| Golgb1 | -0.03703642 |
| Dpy30 | 0.014379501 |
| cdv3 | -0.0016469955 |
| Zfp14 | -0.36352158 |
| Sumf2 | -0.021787643 |
| Pcmt1 | -0.31155396 |
| Yeats4 | -0.28808498 |
| Prpf8 | -0.29952526 |
| Faf2 | -0.30454397 |
| Hpgd | -0.3195057 |
| Eif4g2 | -0.14762592 |
| Cln5 | -0.3010559 |
| Ergic2 | 0.0069332123 |
| Enpp5 | -0.3029256 |
| Zfp957 | -0.3151927 |
| Akr1c18 | -0.28259754 |
| Als2 | 0.045612335 |
