# Supplementary material for: Aire Downregulation Is Associated with Changes in the Posttranscriptional Control of Peripheral Tissue Antigens in Medullary Thymic Epithelial Cells
Source: Front Immunol. 2016 Nov 23;7:526. doi: 10.3389/fimmu.2016.00526 (PMC5120147; doi:10.3389/fimmu.2016.00526)
Supplement: Supplementary file 3 [file table_3.docx]

**Supplemental** Table **3**. **Normalized expression values of miRNAs from Aire knockdown mTECs**

| **miRNAs** | **Normalized expression values** |
| --- | --- |
| miR-425 | 4.046988 |
| miR-1249 | 3.4167247 |
| miR-296-5p | 4.4220486 |
| miR-378 | 1.9191728 |
| miR-361-5p | 3.850038 |
| miR-130b* | 1.5976002 |
| miR-30e* | 3.74614 |
| miR-484 | 7.6348925 |
| miR-149 | 7.1917067 |
| miR-let-7e* | 3.945757 |
| miR-15a* | -5.6704187 |
| miR-671-5p | -5.3821855 |
| miR-432 | -2.2583537 |
| miR-574-3p | -7.1047163 |
| miR-19b-1* | 1.832988 |
