# Supplementary material for: Aire Downregulation Is Associated with Changes in the Posttranscriptional Control of Peripheral Tissue Antigens in Medullary Thymic Epithelial Cells
Source: Front Immunol. 2016 Nov 23;7:526. doi: 10.3389/fimmu.2016.00526 (PMC5120147; doi:10.3389/fimmu.2016.00526)
Supplement: Supplementary file 4 [file table_4.docx]

**Supplemental** Table **4**. **Normalized expression values of mRNAs from Aire knockdown mTECs.**

| **mRNA** | **Normalized expression values** |
| --- | --- |
| Arg1 | -5.0065536 |
| Magi1 | -0.58975697 |
| Thumpd1 | -0.6374302 |
| Ppbp | -5.568082 |
| otub2 | -3.4032164 |
| Prpsap1 | -3.904972 |
| Myom3 | -3.8437443 |
| mtap1a | -1.2852573 |
| Elavl2 | -2.8094406 |
| Pcdh10 | -3.8832574 |
| Cadm4 | -3.5076666 |
| Thbs1 | -3.5088234 |
| Sall3 | -3.4919906 |
| Ephb1 | -3.48573 |
| Foxd3 | -3.8921137 |
| Tex13 | -1.423152 |
| Ino80d | -1.1753273 |
| Slc7a11 | 0.7569051 |
| Pusl1 | -0.82682705 |
