# Supplementary figures and images for: Aire Downregulation Is Associated with Changes in the Posttranscriptional Control of Peripheral Tissue Antigens in Medullary Thymic Epithelial Cells
Source: Front Immunol. 2016 Nov 23;7:526. doi: 10.3389/fimmu.2016.00526 (PMC5120147; doi:10.3389/fimmu.2016.00526)

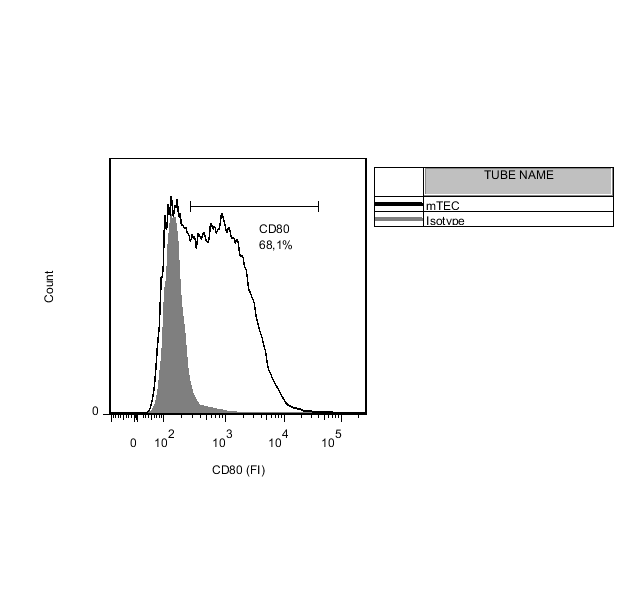

Supplement: Figure S1 — FACS analysis for CD80+ mTEC cells as isolated from thymus by Miltenyi Biotec magnetic beads. [file image_1.png]

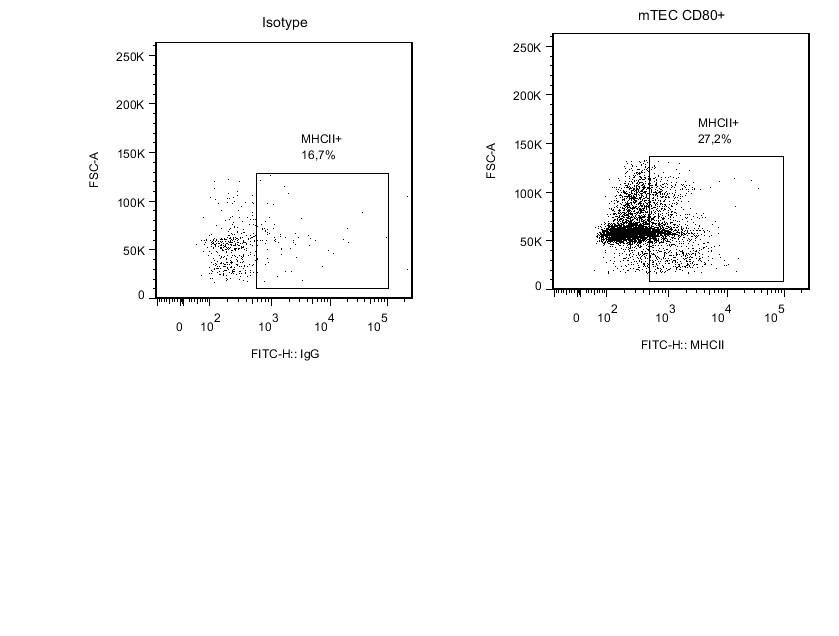

Supplement: Figure S2 — FACS analysis for MHC-II+ mTEC cells as isolated from thymus by Miltenyi Biotec magnetic beads. [file image_2.png]

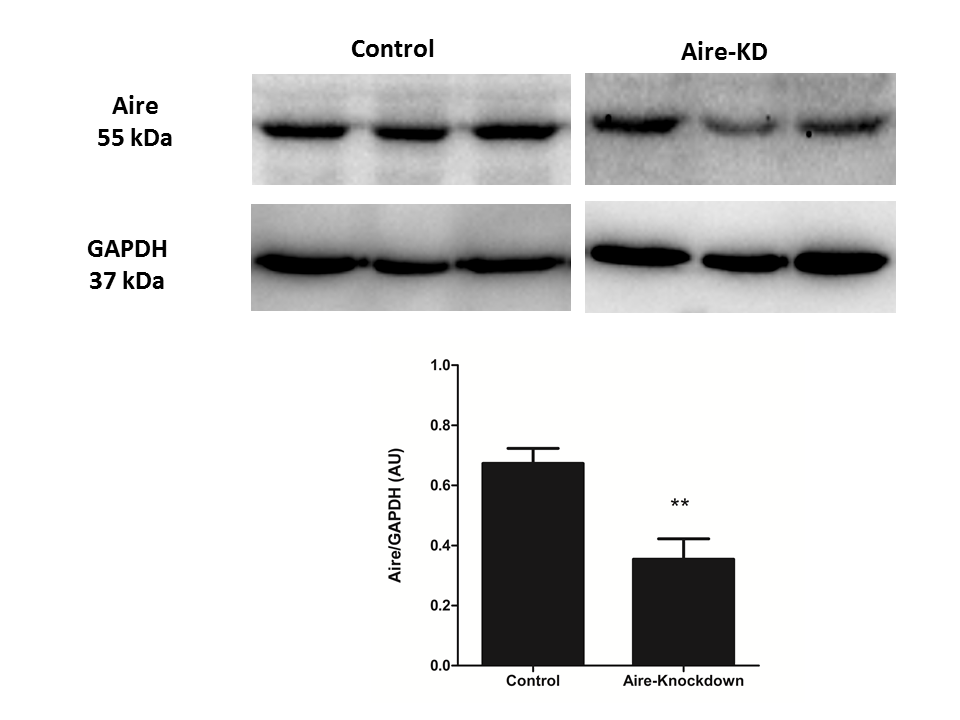

Supplement: Figure S3 — Immunoblotting quantification for AIRE protein in control or Aire-knockdown thymus. Densitometry analysis of immunoblotting shows 50% reduction of AIRE protein expression after Aire-knockdown when compared to the control. GAPDH protein signal was used to normalize loading differences between lanes. Student’s t-test (p < 0.05), KD, knockdown. To measure the AIRE protein levels, PBS (control) or Aire siRNA electro-transfected (Aire knockdown) thymus tissue was excised 48 h after electro-transfection (n = 3 animals per group) and analyzed by immunoblotting (western blotting). The thymic stroma was homogenized in extraction buffer plus a protease inhibitor cocktail (Sigma-Aldrich, St. Louis, MO, USA). The total thymic stromal proteins (20 μg protein/lane) were resolved on by 12% sodium dodecyl sulfate polyacrylamide gel electrophoresis (SDS-PAGE) and electro-transferred (electro-blotting) to a polyvinylidene difluoride (PVDF) membrane (Amersham Hybond P 0.2, GE Healthcare Life Sciences). The membranes were blocked with 5% bovine serum albumin in 1× Tris-buffered saline (pH 7.9) with 0.05% Tween 20 (BSA/TBST) for 3 h. The membranes were incubated overnight at 4°C with 1:500 dilution of an anti-AIRE 1 (D-17): Sc-17986 goat polyclonal primary antibody (Santa Cruz Biotechnology Inc., Santa Cruz, CA, USA), followed by incubation with 1:10,000 dilution of a horseradish peroxidase (HRP)-conjugated donkey anti-goat IgG Sc-2020 secondary antibody (Santa Cruz Biotechnology) for 1 h at room temperature. To control for lane loading, the membranes were probed with a goat anti-GAPDH antibody (Cell Signaling, Danvers, MA, USA; 1:10,000 dilution) after washing with stripping buffer. The membranes were developed using the peroxidase substrate for enhanced chemiluminescence (ECL) (Millipore, Billerica, MA, USA). Gel documentation and signal quantification were performed using bio-imaging analysis on a molecular imager apparatus (ChemiDoc XRS System, Bio-Rad, Richmond, CA, USA). The image [file image_3.tif]
